# sEMG Activation of the Flexor Muscles in the Foot during Balance Tasks by Young and Older Women: A Pilot Study

**DOI:** 10.3390/ijerph16224307

**Published:** 2019-11-06

**Authors:** Monika Błaszczyszyn, Agnieszka Szczęsna, Katarzyna Piechota

**Affiliations:** 1 Faculty of Physical Education and Physiotherapy, Opole University of Technology, Prószkowska 76, 45-758 Opole, Poland; k.piechota@po.edu.pl; 2 Institute of Informatics, Silesian University of Technology, Akademicka 16, 44-100 Gliwice, Poland; agnieszka.szczesna@polsl.pl

**Keywords:** balance tasks, dorsal flexor, plantar flexor, surface electromyography

## Abstract

Objective: In this publication, we suggest that young adults and seniors use various defense mechanisms to counteract loss of balance. One of the hypotheses is the change in the coordination of antagonistic muscle groups, especially within the ankles. In this study, we tried to determine if there is a relationship between the condition from resilient, to pre-frail, to frail and the ability to maintain balance during free standing and balance tasks. The aim of the study was to define the importance of muscle activity in the ankle joint, dorsal flexor of the foot for the following: tibialis anterior (TA), plantar flexor of the foot gastrocnemius medialis (GM), gastrocnemius lateralis (GL), and peroneus longus (PER), during balance tasks with eyes open (EO) and closed (EC). We hypothesized that there are differences in the activity and co-activation of the tested muscles in young and older women, which may indicate an increased risk of falls and walking disorders. Materials and methods: A group of 20 women qualified for the study. The group was divided into two subgroups, young (G1) and elderly women (G2). The aim of the study was to define the importance of muscle activity in the ankle joint, dorsal flexor of the foot for the following: tibialis anterior (TA), plantar flexor of the foot gastrocnemius medialis (GM), gastrocnemius lateralis (GL), and peroneus longus (PER), during balance tasks with eyes open (EO) and closed (EC). Results: In this study, we observed significant differences between groups in the maximum and mean values of electromyography activity (EMG) activation of the examined muscles on different types of surfaces and with open and closed eyes. Older women generated higher values of EMG activation in all muscles except the gastrocnemius medialis muscle. The results were significant for co-activation at rest for muscles as follows: tibialis anterior and gastrocnemius medialis with eyes closed (*p* = 0.01) and peroneus and gastrocnemius lateralis at rest with eyes open (*p* = 0.03), eyes closed (*p* = 0.04), and on a foam (*p* = 0.02). The sEMG amplitude of the tested muscles means that agonist muscle activity changed relative to antagonistic muscle activity. Conclusions: Activation of sEMG and coordination of ankle muscles during balance tasks change with age. It can be hypothesized that assessment of balance during free standing and equivalent tasks can predict the state of frailty, after taking into account other physiological variables that are believed to affect balance control.

## 1. Introduction 

Frailty syndrome (FS) is characterized by a high susceptibility to adverse health effects, such as disability, weakness, falls, hospitalization, and mortality. It is considered to be a dynamic condition characterized by a set of symptoms that can improve or worsen over time. Many authors believe that it is indirectly related to the aging process, although it is currently unclear whether, and to what extent, aging and FS should be differentiated [1]. There is still no clear definition of FS or a universal clinical evaluation. One of the biggest problems concerns the characteristic features that could distinguish FS from the progressive signs of "normal" aging. As clinical studies show, not all older adults become frail and even at a very advanced age this percentage remains below 50% [2]. Although the musculoskeletal system is an important therapy target, FS is thought to be more complex than sarcopenia and dynopenia alone. However, strength, endurance, and balance training are still considered the best strategies to improve walking ability, balance, strength performance, and reduce the frequency of falls in elderly [3]. 

A report on a research project conducted by a group of Italian and American geriatricians recommended that the following factors should be included in the assessment of FS in physiological areas: mobility, balance, muscle strength, walking, cognitive function, nutrition, and physical activity [4]. Fried’s model is the best known and widely used tool for detecting weak points and identifying weakness with sarcopenia as a key pathophysiological feature. Additionally, a strength of Fried’s model is that it has been approved as a predictor of adverse results in large epidemiological studies [2]. Studenski et al. developed the CGIC-PF (Clinical Global Impression of Change in Physical Frailty) instrument, which includes: mobility, balance, strength, endurance, nutrition, neuromotor performance, medical care, use of healthcare, appearance, well-being, subjective assessment of health, ADL (activity of daily living), emotional state, and social status [5]. One of the recurring factors in FS assessment is balance during free standing, which involves the complex interactions of many postural control systems that can be degraded. It consists of the interaction of sensory, nervous, and musculoskeletal systems [6]. In response to the decline of the neuromusculoskeletal system, a compensatory increase in postural muscle activities occurs in the elderly during upright standing. We highlighted a positive correlation between CoP displacements and the relative electromyography activity (EMG) of the triceps surae during increasingly difficulty postural tasks in the elderly, and therefore assume the mechanical contributions of muscles mobilizing ankle joint during quiet standing [7,8]. 

In our research, we wanted to check the relationship between balance and the process of human aging. 

The following factors are mentioned in the literature as causing an increased risk of falling in the group of elderly people: visual impairment, drug intake, deficiency in the strength of lower limbs, increased time spectrum, and variability of gait, and impaired balance efficiency [9,10]. The loss of vestibular, visual, somatosensory, and neuromuscular function caused by aging leads to a deterioration of postural control when performing tasks of standing balance in a group of the elderly [11]. The available evidence suggests that young adults and seniors use different strategies to adapt to increasing body sway when standing, which may be caused by the change in the cooperating muscular antagonist and different patterns of coordination of the ankle muscles [12]. Proprioception provides information about the static and dynamic elements of orientation and body posture. Information from the lower limb, especially from the ankle joint, is extremely important. This sensory input can be measured experimentally using, for example, a posturographic platform, muscle vibrations, or the structure of the base [13]. Sensory visual input can be easily modified by opening and closing the eyes, which causes changes in postural stability [14]. Ultrasound, EMG, inertial sensors, and load cells have been proven to be very reliable instruments in the architectural, functional, and kinetic analysis of the dorsiflexion of the foot [15]. Changes in muscle activation are required to maintain balance, but must occur at the appropriate moment.

Human standing balance is continuously challenged by gravity, which imposes a negative stiffness on the upright equilibrium posture. This stiffness must be compensated in order to maintain an upright stance. The ankle muscle and tendon structures provide stiffness at multiple levels. Separating the reflexive and intrinsic contributions to the overall ankle stiffness during standing could possibly give insight into neuromuscular disorders and could help in the assessment of balance control, in a clinical setting. These results suggest that intrinsic ankle-foot stiffness alone is insufficient to maintain upright balance. Consequently, either changes in ankle muscle activation are required, or another method of control [16]. The sEMG (surface electromyography) method, in connection with other biomechanical methods, indicates important information about muscle activity during performance of different types of activities with changeable load, angle positioning in joints, and changeable speed [17].

This study concerns relationships between the condition from resilient, to pre-frail, to frail and the ability to maintain balance during free standing and balance tasks. The aim of the study is to define the importance of muscle activity in the ankle joint, dorsal flexor of the foot for the following: tibialis anterior (TA), plantar flexor of the foot gastrocnemius medialis (GM), gastrocnemius lateralis (GL), and peroneus longus (PER), during balance tasks with eyes open (EO) and closed (EC). We have hypothesized that there are differences in the activity and co-activation of the tested muscles in young and older women, which may indicate an increased risk of falls and walking disorders.

## 2. Methods

A group of 20 women qualified for the study. The group was divided into two subgroups as follows: the first group, G1, were young women (22 ± 1.8 years, 165.7 ± 6.1cm, 53.3 ± 5.1 kg, and BMI (body mass index) 20.83 ± 2.8 kg/m^2^) and the second group, G2, were elderly women (69 ± 4.8 years, 155.7 ± 3.4 cm, 66.3 ± 6.7 kg, BMI 25.97 ± 2.69 kg/m^2^, and MMSE (Mini-Mental State Examination) 27.9 ± 2.1). In addition, older women were diagnosed with frailty according to Fried’s criteria as follows: no weight loss was observed in the subjects; none of them reported exhaustion; physical activity, measured by the IPAQ short Polish version questionnaire [18], none of the women continued their professional work and the average physical activity was 1200 METday/week (minimum 672 METday/week, maximum 1728 METday/week); the average walking time was 8.3 ± 1.7 s; and muscular strength, measured with a dynamometer manual, was 18.3 ± 1.6 kg on average. The study was performed on the dominant leg. All participants declared the right leg as dominant. The criterion of participation in the study were the following: the lack of lower limb injuries, neuromuscular system diseases, and fall history, as well as no sight or hearing disorders, and no problems with keeping balance. Participants did not report any medication intake and health impairments that could affect balance and muscle activity testing. Seniors with diabetes, untreated hypertension, glaucoma, endoprosthesis, stroke, eczema, and beta-blocker users were not included in the study.

## 3. Procedures

A trained researcher recorded baseline measurements. The first step was an interview which included: (1) health condition, (2) demographic variables (age and sex), (3) chronic conditions (mental diseases, cardiovascular alterations, rheumatoid arthritis, diabetes mellitus type 1 and 2, metabolic syndrome, obesity, neurological problems, musculoskeletal alterations, and vascular illness), (4) MMSE (Mini-Mental State Examination), and (5) Fried’s criteria.

**Figure 1:**
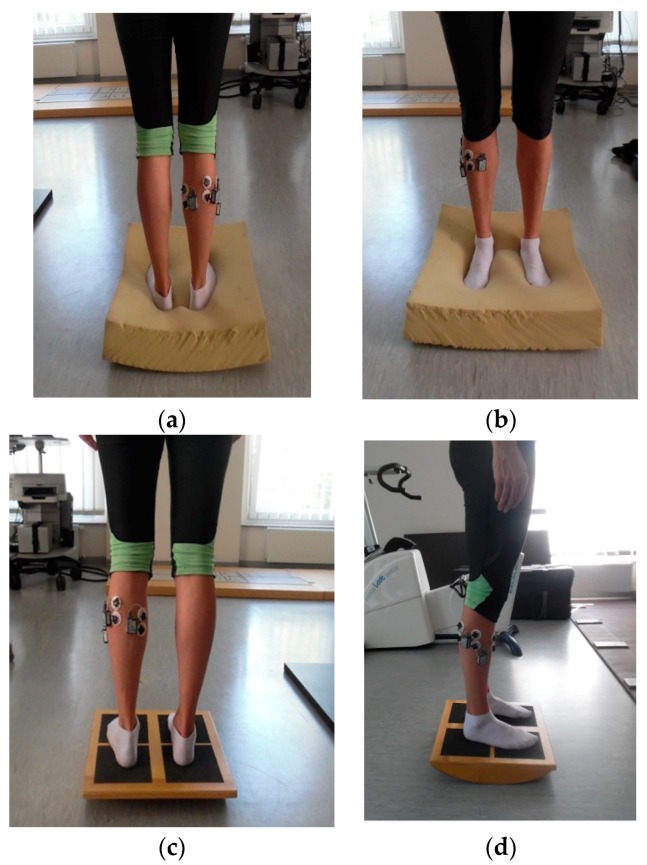
Illustration of EMG signal recording process. Note: the picture shows one of the young woman: (**a**) standing on a foam, plantar flexor of the foot gastrocnemius medialis (GM), gastrocnemius lateralis (GL), (**b**) standing on a foam, dorsal flexor of the foot tibialis anterior (TA), (**c**) standing on a “seesaw”, plantar flexor of the foot gastrocnemius medialis (GM), gastrocnemius lateralis (GL), (**d**) standing on a “seesaw”, plantar flexor of the foot peroneus longus (PER).

The subjects took off their footwear, and after a researcher assessed height and weight, the body mass index (BMI) was calculated from height (m) and weight (kg^2^), applying the Quetelet’s equation as BMI = weight/height^2^. Finally, the EMG measurements were recorded at six time-normalized trials, preceded by a training trial. The duration of each attempt was 20 s on all types of surface with eyes open (EO) and closed (EC), with a 5 s break for a command, “eyes closed”. The participants, one by one, performed standing on a hard stable surface with EO and EC, next, standing on a foam with dimensions of 50 × 50 cm, height 20 cm, density 40 kg/m^3^ with EO and EC. The distance between feet was 30 cm. The last activity was standing on a seesaw, in a position with the possibility of moving forwards and backwards, with EO and EC (Figure 1). The participants received a command to hold a stable posture, without raising feet from the ground, no visual goal was defined, and the participants looked straight ahead.

This study was approved by the local ethics committee and fulfilled the criteria of the Helsinki declaration [19]. The scope and goal of this study were approved by the Bioethics Committee of the Chamber of Physicians (Resolution No. 237 of 13 December 2016). All participants signed an informed written consent prior to the start of the study. System EMG produced by Noraxon (Scottsdale, AZ, USA), which registers muscle activity, was used as a data acquisition tool. Before placing the electrodes, the skin was carefully shaved and cleaned using an abrasive cleaner and alcohol swabs to reduce impedance. The placement and location of the surface electrodes conformed to the recommendations by SENIAM (Surface EMG for noninvasive Assessment of Muscles) [20].

Electrodes were carefully positioned on the belly of each muscle, parallel to the orientation of muscle fibers with an interelectrode distance of 25 mm. Disposable electrodes Bio-Lead-Lok B R-LFO-300 (Bio-Lead-Lok B, Józefów, Poland) were used with a round pickup area. The data analysis was performed using the MyoResearch XP MT 400 program (Noraxo, Scottsdale, AZ, USA). The sampling rate was 1000 Hz. The root mean square (RMS) values of EMG signals were calculated for consecutive segments of 50 ms. We defined outliers as values outside the interval mean ± 3 SD [21].

**Figure 2:**
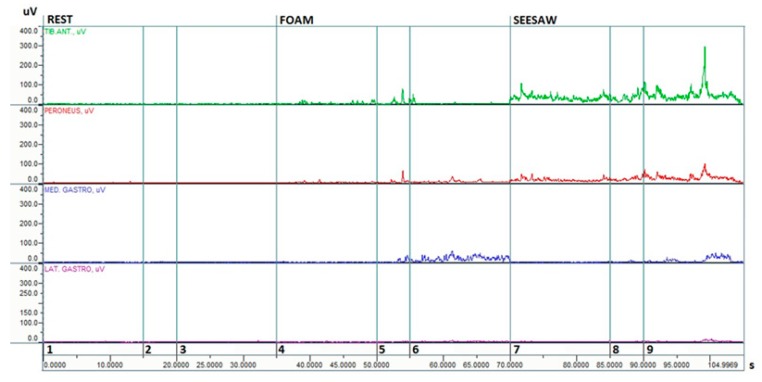
Example electromyography (EMG RMS) responses during trials for a young woman: age 21, height 165 cm, weight 55 kg. Note: 1. rest and eyes open; 2, break; 3, rest and eyes closed; 4, foam and eyes open; 5, break; 6, foam and eyes closed; 7. seesaw and eyes open; 8, break; and 9, seesaw and eyes closed.

**Figure 3:**
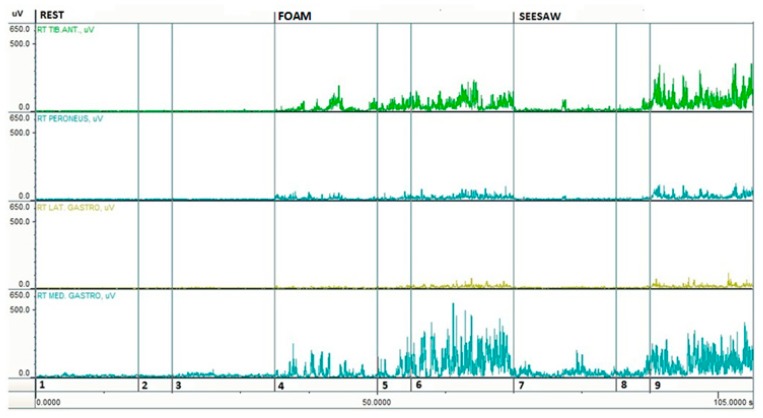
Example electromyography (EMG RMS) responses during trials of an older woman: age 67, height 160 cm, and weight 65 kg. Note: 1, rest and eyes open; 2, break; 3, rest and eyes closed; 4, foam and eyes open; 5, break; 6, foam and eyes closed; 7, seesaw and eyes open; 8, break; and 9, seesaw and eyes closed.

Potential changes in muscle coordination between the tibialis anterior and gastrocnemius medialis activity were examined using a co-activation ratio of the EMG RMS amplitudes (Figure 2 and Figure 3) [22,23]. 

Thus, a decrease in this ratio means that there was less co-activation achieved by either an increase in tibialis anterior RMS amplitude or a decrease in RMS gastrocnemius medialis amplitude.

## 4. Data Analysis

A normality distribution analysis was carried out by the Shapiro–Wilk’s test, considering a normal distribution when *p* > 0.05. Demographic characteristics such as age, height, weight, and BMI were described. Considering the quantitative values, the mean and standard deviation (SD) were applied to the dataset. 

Next, the normality analysis for mean and maximal RMS values of EMG responses was carried out using the Kolmogorov–Smirnov test, the results were not significant (*p* > 0.05). Nonparametric Mann–Whitney U-test was used for comparisons of mean and maximal (peak) RMS EMG values of participants between group G1 and G2 (Table 1). The same statistical test was used to check the statistical differences between the co-activation coefficient values in group G1 and G2 (Table 2). Compared values were calculated for 50 ms EMG data signal segments. In all tests the *p*-values less than 0.05 were statistically significant. 

In addition, the N-way analysis of variance ANOVA was used for testing the effects of multiple factors on the mean of the data. The following four grouping variables were defined: X1 (group G1 and G2); X2 (muscles: (TA) tibialis anterior, (PER) peroneus, (GM) gastrocnemius medialis, and (GL) gastrocnemius lateralis); X3 (eyes open and eyes closed); and X4 (surface: rest, foam, and seesaw).

All statistical analyses were carried out using Matlab 2016 software. 

## 5. Results

The results of p-value (*p* < 0.05) of multiway analysis of variance (ANOVA) are small enough to conclude that the mean responses are significantly different for the defined four factors in the analysis of the mean and maximal values of EMG RMS amplitudes.

All results described below are based on the results of the the nonparametric Mann–Whitney U-test.

On the basis of the data listed in Table 1, significant differences between groups for the maximum and mean values of sEMG activation of the examined muscles on different types of surfaces and with open and closed eyes were observed. The observations showed significant differences in sEMG activation. Older women generated higher values of sEMG activation in all muscles except the gastrocnemius medialis muscle.

## 6. Co-Activation Ratio

The co-activation ratio was defined as antagonistic muscle activity (tibialis anterior and peroneus maximum RMS amplitude) divided by agonist muscle activity (gastrocnemius medialis and lateralis maximum RMS amplitude). A decrease in the ratio shows that there was greater agonistic activity or less antagonistic activity.

On the basis of the data listed in the Table 2, result were significant for the co-activation co-efficient, at rest for the muscles, tibialis anterior and gastrocnemius medialis with eyes closed (*p* = 0.01), as well as peroneus and gastrocnemius lateralis at rest with eyes open (*p* = 0.03), eyes closed (*p* = 0.04), and on a foam (*p* = 0.02). The RMS amplitude of the muscles tested means that agonistic muscle activity has changed relative to antagonistic muscle activity.

## 7. Discussion

This study during balance tasks showed an increase in sEMG amplitude (Table 1) and a change in co-activation of the dorsal flexor of the foot and plantar flexor of the foot (Table 2). For the co-activation ratio, which was calculated as antagonistic muscle activity divided by agonist muscle activity, a decrease in the ratio means that there is greater agonistic activity or less antagonistic activity opposing the movement [24,25]. The results highlight that the combined effect of age and obesity is responsive to an increase of postural control alterations. It is associated with higher muscle activities, especially at the PF level, however, it is interesting that decreases in antagonistic sEMG activity have also been observed in short-term training studies [26,27]. In the elderly, the postural control system is increasingly affected by the reduction of muscle strength and deterioration of sensory integration which causes a decrease in acuity in perception of joint angular position and movement and an increase in the latency of postural responses [28]. To compensate for the deterioration of the postural control system, older adults show increased co-activation of the ankle muscles. In addition, Benjuya et al. [29] concluded that elderly people stiffened and froze the lower limbs to counteract the increase in body rolling during difficult balance tasks. According to Tinetti et al. [30], the most important limitation of the biomechanical balance is the size and quality of the base of foot support. Any restrictions related to muscle strength, range of joint mobility, pain or foot control could affect the ability to maintain balance. It is illustrated, most importantly, by the increased activation of sEMG of the muscle and increased muscular co-activation in seniors. In addition, there is evidence that older people have impaired modulatory reflexes and muscle response characteristics, which indicates limited ability to adapt to changing postural conditions. The strategy of simultaneous activation of antagonists may result from the potential weakening of proprioceptive information or the limited ability to generate muscle strength in older people [31].

The obtained results indicated the differences occurring in the muscle activity of agonists in ratio to antagonists of the ankle This can be explained by the fact that older people strive to increase the plane of support which is observed by increased sEMG activation in the lateral muscle group (PER and GL) (Table 2). On the one hand, it provides them with posture that has greater stability, whereas, on the other hand, it probably deprives defense mechanisms in a situation of changing the support plane, further exposing older people to greater body asymmetry and increased susceptibility to disturbing the balance of the body as a result of unstable surfaces [32,33]. The effect of these changes is the lack of quick and effective adjustments in postural control to changing surface conditions, which consequently leads to balance disorders and falls [34]. It has been shown that elderly may let their muscles be in a high-activated state, thereby increasing muscle co-activation to counteract the decrease in relative force and maintain stability. Consequently, to maintain balance, the elderly need to counteract forward instability by increasing plantar flexor muscle activity to compensate for decreased force production capacities by increasing muscle co-activation, however, strong muscle activities increase the risk of excessive energy expenditure, resulting in early fatigue and a higher risk of falls [35]. It seems likely that increased co-activation during balance tasks is a more compensatory strategy to counteract the effects of aging, related to muscle weakness or fatigue. It seems particularly true for the tibialis anterior muscle. The neuromuscular properties (e.g., muscle contraction) of the TA muscle undergo significant changes with age and are additionally considered to be the main factor contributing to the decline in sEMG frequency. However, due to the substantially lower muscular volume of the TA as compared with the muscle group triceps surae, even a high level of co-activation does not necessarily reflect a balanced biomechanical state of the muscle [36]. Many geriatricians believe that clinically, full-size FS means a sharp decline, from which the overall health of many older adults deteriorates with a decrease in the likelihood of recovery. This view of FS is consistent and was created in the literature by Fried, Walston and colleagues [2]. The FS index requires additional clinical evaluation, which will allow more precise determination of FS risk factors. Currently, they are based on a single scale and do not capture multiscale physiological disorders [7]. In future studies, it is worth considering standing in footwear, because as Roca-Dols et al. [37,38] suggest, muscle activity patterns may differ during barefoot standing as compared with standing in shoes, which was not included in our study. Our study was limited to checking the activity of the surface muscles of the calf, not taking into account the impact on plantar intrinsic foot muscles (PIFMs), which can contribute to a reduction of the stress on the plantar in static states [39].

## 8. Limitations

This study did not take into account the kinematic parameters and the EMG activation of muscles of other lower limb and trunk segments. Further cross-sectional and long-term studies should be performed, covering a larger group of respondents of both genders. More work is needed to check if balance during free standing and equivalent tasks can predict the continuum from resilient, to pre-frail, to frail. 

## 9. Conclusions

Activation of sEMG and the coordination of ankle muscles during balance tasks change with age. It can be hypothesized that assessment of balance during free standing and equivalent tasks can predict the state of frailty, after taking into account other physiological variables that are believed to affect balance control.

## Figures and Tables

**Table 1:** Median of mean and maximum values of RMS amplitude of the examined muscles (µV) in the elderly and young participants on various types of surfaces.

**Rest**	**TA eo**	**TAec**	**PER eo**	**PER ec**	**GM eo**	**GM ec**	**GL eo**	**GL ec**
*p*	**<0.01**	**<0.01**	**<0.01**	**<0.01**	0.42	0.96	**0.02**	**<0.01**
Mean G1	2.63	2.92	3.72	4.04	5.29	8.41	4.14	3.93
Mean G2	7.05	7.71	12.16	10.86	8.22	7.53	6.32	7.11
*p*	**<0.01**	**<0.01**	**<0.01**	**<0.01**	0.42	0.90	0.14	**0.03**
Max G1	5.41	8.22	10.04	9.98	14.30	37.40	10.01	10.24
Max G2	58.23	87.11	38.27	32.65	26.07	39.76	19.82	15.65
**Foam**	**TA eo**	**TAec**	**PER eo**	**PER ec**	**GM eo**	**GM ec**	**GL eo**	**GL ec**
*p*	**<0.01**	**0.01**	**<0.01**	**<0.01**	0.05	0.47	**<0.01**	**0.02**
Mean G1	3.30	10.95	4.80	13.29	8.10	18.25	5.12	8.70
Mean G2	18.28	44.99	21.66	42.81	22.92	37.39	9.79	15.36
*p*	**<0.01**	**<0.01**	**<0.01**	**<0.01**	0.73	0.73	**0.02**	0.52
Max G1	17.90	87.05	18.35	53.60	47.95	165.50	14.05	28.15
Max G2	99.99	251.17	61.26	155.70	58.65	112.59	29.76	41.54
**Seesaw**	**TA eo**	**TAec**	**PER eo**	**PER ec**	**GM eo**	**GM ec**	**GL eo**	**GL ec**
*p*	**<0.01**	**0.02**	**<0.01**	**<0.01**	0.18	0.73	**0.03**	**0.02**
Mean G1	6.16	41.15	9.30	29.10	9.68	51.30	5.01	11.05
Mean G2	40.89	80.19	42.14	55.93	16.17	37.30	11.37	19.19
*p*	**<0.01**	0.08	**<0.01**	0.05	0.10	0.90	**0.03**	0.24
Max G1	28.80	215.50	36.95	109.00	44.30	169.00	13.05	59.80
Max G2	196.81	334.83	108.06	243.42	90.76	144.05	41.98	111.82

Notes: *p* < 0.05; G1, young women; G2, elderly women; Max, average peak value; Mean, average value; TA, tibialis anterior; PER, peroneus; GM, gastrocnemius medialis; GL, gastrocnemius lateralis; eo, eyes open; and ec, eyes closed.

**Table 2:** Test results (*p*-value) of the co-activation ratio between specified muscles groups, based on young and elderly women data on different types of surfaces.

Surface	TA/GM eo	TA/GM ec	PER/GL eo	PER/GL ec
Rest	0.08	0.01	0.03	0.04
Foam	0.06	0.10	0.22	0.02
Seesaw	0.05	0.12	0.19	0.49

Notes: *p* < 0.05; G1, young women; G2, elderly women; TA, tibialis anterior; PER, peroneus; GM, gastrocnemius medialis; GL, gastrocnemius lateralis; eo, eyes open; and ec, eyes closed.
